# Supplementary material for: Improvement of Interlayer Adhesion and Heat Resistance of Biodegradable Ternary Blend Composite 3D Printing
Source: Polymers (Basel). 2021 Feb 27;13(5):740. doi: 10.3390/polym13050740 (PMC7957628; doi:10.3390/polym13050740)
Supplement: Supplementary file 1 [file polymers-13-00740-s001.pdf]

Improvement of Interlayer Adhesion and Heat Resistance of Biodegradable Ternary Blend Composites 3D Printing

Wattanachai Prasong [1], Akira Ishigami [1,2], Supaphorn Thumsorn [2], Takashi Kurose [2] and Hiroshi Ito [1,2,*]

[1] Graduate School of Organic Materials Science, Yamagata University, 4-3-16 Jonan, Yonezawa, Yamagata 992-8510, Japan; wattanachai.pra@gmail.com (W.P.); akira.ishigami@yz.yamagata-u.ac.jp (A.I.)
[2] Research Center for GREEN Materials and Advanced Processing (GMAP), 4-3-16 Jonan, Yonezawa, Yamagata 992-8510, Japan; thumsorn@yz.yamagata-u.ac.jp (S.T.); takashi.kurose@yz.yamagata-u.ac.jp (T.K.)
* Correspondence: ihiroshi@yz.yamagata-u.ac.jp; Tel.: +81-238-26-3081

Supplementary Data

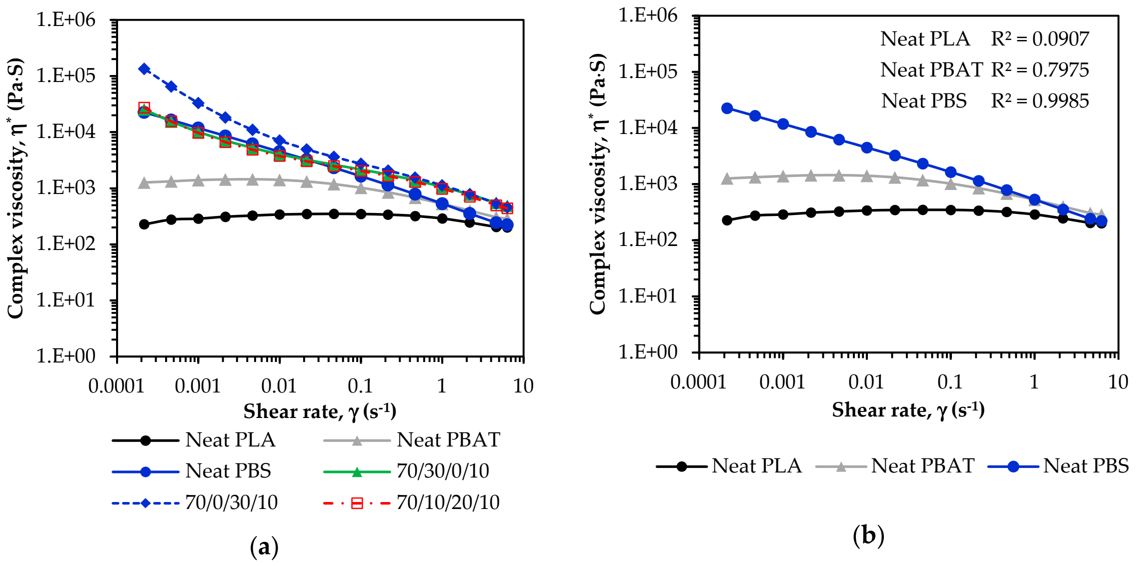

Figure S1. (a) Complex viscosity as a function of shear rate of neat polymers and PLA/PBAT/PB/nano talc composites at 210 °C; (b) Power regression of power law index for shear thinning behavior of neat polymers.

The power-law model for determining the shear thinning behavior [27, 36, 42].

$$\eta = K\dot{\gamma}^{n-1} \quad \text{(S1)}$$

Where η is the shear viscosity at shear rate of 0.2 s^{-1}, K is the consistency index, $\dot{\gamma}$ is the shear rate, and n is the power-law index.

Table S1. Determination of flow behavior of neat polymers and PLA/PBAT/PBS/nano talc composites.

Polymer	η at $\dot{\gamma}$ 0.01 s^{-1} (Pa·s)	η at $\dot{\gamma}$ 0.2 s^{-1} (Pa·s)	n[1]	K[1] (Pa·s)
PLA	340	339	0.98	274
PBAT	1412	847	0.85	536
PBS	4482	1143	0.55	535
70/30/0/10	4032	1753	0.65	953
70/0/30/10	7075	2069	0.49	983
70/10/20/10	3758	1648	0.64	873

[1] Analyzes by power regression.

The estimation of blend composition in the vicinity of phase inversion [36, 42].

$$\frac{\eta_1}{\eta_2} = \frac{\varphi_1}{\varphi_2} \tag{S2}$$

Where η_1 is the viscosity of polymer 1, η_2 is the viscosity of polymer 2, φ_1 is the volume fraction of polymer 1, and φ_2 is the volume fraction of polymer 2. If the ratio of $\frac{\eta_1}{\eta_2} > \frac{\varphi_1}{\varphi_2}$, then polymer 2 will be the continuous phase [35].

Table S2. The ratio of viscosities and the volume fractions of polymers for predicting phase inversion in binary polymer blends.

Binary Blends		Matrix	Dispersed phase	Phase inversion
PLA/PBAT (70/30)	$\frac{\eta_1}{\eta_2} < \frac{\varphi_1}{\varphi_2}$	PLA	PBAT	PBAT > 71.5 %
PLA/PBS (70/30)	$\frac{\eta_1}{\eta_2} < \frac{\varphi_1}{\varphi_2}$	PLA	PBS	PBS > 77.5 %
PBAT/PBS (70/30)	$\frac{\eta_1}{\eta_2} < \frac{\varphi_1}{\varphi_2}$	PBAT	PBS	PBS > 57.5 %
PBS/PBAT (70/30)	$\frac{\eta_1}{\eta_2} < \frac{\varphi_1}{\varphi_2}$	PBS	PBAT	PBS > 42.5 %

Remark: Shear viscosity of η_1 and η_2 at shear rate 0.2 s^{-1} [23-24, 36].

The Harkin's spreading equation can be used to predict the type of wetting phenomenon in ternary blends [20, 23-24, 37-41].

$$\lambda_{ABC}\ or\ \lambda_{BC} = \gamma_{AC} - \gamma_{AB} - \gamma_{BC} \tag{S3}$$

$$\lambda_{ACB}\ or\ \lambda_{CB} = \gamma_{AB} - \gamma_{AC} - \gamma_{BC} \tag{S4}$$

$$\lambda_{BAC}\ or\ \lambda_{AB} = \gamma_{BC} - \gamma_{AB} - \gamma_{AC} \tag{S5}$$

Where λ is the spreading coefficient, γ is the interfacial tensions for the polymer pairs and sub-indices refer to each phase in the mixture. A is PLA, B is PBAT and C is PBS.

Surface tension and interfacial tension from the harmonic mean equation for calculation of interfacial tension [23].

$$\gamma = \gamma_1 + \gamma_2 - \frac{4\gamma_1^d\gamma_2^d}{\gamma_1^d+\gamma_2^d} - \frac{4\gamma_1^p\gamma_2^p}{\gamma_1^p+\gamma_2^p} \tag{S6}$$

Where γ is surface tension, γ^d is the dispersive contribution of surface tension, and γ^p is the polar contribution of surface tension.

Table S3. Surface tensions of PLA, PBAT and PBS and interfacial tensions of polymer blends [23].

Polymer (Phase)	γ	γ^d	γ^p	γ of polymer blends	Interfacial tension (mN/m)
PLA (A)	38.8 ± 0.2	30.3 ± 0.2	8.5	$\gamma_{PLA/PBAT}$	0.08 ± 0.01
PBAT (B)	41.4 ± 0.3	32.3 ± 0.2	9.1 ± 0.1	$\gamma_{PLA/PBS}$	0.33 ± 0.03
PBS (C)	43.6 ± 0.4	33.1 ± 0.3	10.5 ± 0.1	$\gamma_{PBAT/PBS}$	0.11

[1] γ, γ^d and γ^p from the reference [23].

Table S4. The Harkin's spreading coefficient of ternary blend PLA/PBS/PBAT.

λ	Spreading coefficient (mN/m)
$\lambda_{PLA/PBAT/PBS}$	0.14 ± 0.02
$\lambda_{PLA/PBS/PBAT}$	-0.36 ± 0.02
$\lambda_{PBAT/PLA/PBS}$	-0.31 ± 0.03
